# Comparison of the agreement between WeChat-based self-administered and interviewer-administered data on infant and young child feeding in China: A test-retest study

**DOI:** 10.7189/jogh.12.11004

**Published:** 2022-06-20

**Authors:** Aihua Liu, Jian Zhang, Qiong Wu, Yanfeng Zhang, Michelle van Velthoven

**Affiliations:** 1 Department of Integrated Early Childhood Development, Capital Institute of Pediatrics, Beijing, China; 2 Nuffield Department of Primary Care Health Sciences, University of Oxford, Oxford, UK

## Abstract

**Background:**

Measuring infant and young child feeding (IYCF) indicators is important in evaluating child health programs and making evidence-based decisions. With Internet and new media rapidly developing, communication apps such as WeChat (the most popular mobile social media platform in China) are widely used and can potentially be used as an alternative way to collect infant and young child feeding information. This study compares data agreement between a WeChat-based self-administered and an interviewer-administered survey on infant and young child feeding information.

**Methods:**

We recruited 297 mothers of children aged 6-23 months in Fenxi County, Shanxi Province, China. Using the Test-Retest method, we first collected data through a self-administered survey using a WeChat-based electronic questionnaire and asked 36 questions on breastfeeding and complementary feeding knowledge, practices, and information sources. We then conducted an interviewer-administered survey using the same questionnaire and compared the data agreement between the two survey methods during the same day. Cohen's kappa score (κ) and intraclass correlation coefficients (ICC) were used for data agreement analysis for all 36 questions and six key IYCF indicators. The McNemar test was used to identify differences between the two survey methods for the six key indicators.

**Results:**

There was substantial or almost perfect agreement for 33 questions (κ/ICC>0.60), and slight or fair agreement for the other 3 questions (κ/ICC<0.40). Agreement of all six key IYCF indicators was substantial or almost perfect (κ = 0.78-0.94), while two indicators showed statistical differences between the two survey methods (*P* = 0.03 for “Minimum meal frequency” and *P* = 0.001 for “Minimum accepted diet”). Analysis of reasons for inconsistencies showed that 43.6% of all the inconsistencies were not caused by the self-administered survey method. The cost of the interviewer-administered survey was much higher than that of the self-administered survey: ¥45.9 (US$6.8) vs ¥19.7 (US$2.9) per questionnaire.

**Conclusions:**

The WeChat-based self-administered method can be used for future data collection of infant and young child feeding information in China. Most of the questions and key indicators showed very good agreement without statistical differences between the two methods.

Nutrition in early life is crucial to children's growth and development, but malnutrition in infants and young children is very common in low- and middle-income countries (LMICs) [1]. Inappropriate feeding can lead to malnutrition, allergies, obesity, iron deficiency anaemia, and other health problems, and can have an irreversible impact on the growth of children [2]. The World Health Organization (WHO) and the United Nations Children's Fund (UNICEF) suggest that exclusive breastfeeding of infants should be carried out from birth to six months, complementary food should be added at the end of six months, and breastfeeding should be continued until children are at least two years old [3]. Recent data indicate that the exclusive breastfeeding rate of infants under six months of age was 41% worldwide [4], and 37% in LMICs [5]. In China, the exclusive breastfeeding rate was only 29.8% in 2018 [6], far below the national target of 50%. For complementary feeding, only one in six children aged 6-23 months in LMICs were fed the minimum acceptable diet [7]. In China, the proportions of children who met minimum dietary diversity, minimum meal frequency and minimum acceptable diet were 53.7%, 69.1%, and 25.1% in 2013, respectively [8]. Therefore, China urgently needs to improve its infant and young child feeding (IYCF) practices.

The coverage of IYCF interventions needs high-quality measurements to effectively track progress and make evidence-based decisions [9]. In 2008, WHO and UNICEF jointly released “Indicators for assessing infant and young child feeding practices” [10]. The accompanying interviewer-administered household survey questionnaire was published in 2010 [11]. As a full range of globally recommended feeding indicators, the IYCF indicators and questionnaires have been used in studies worldwide [7]. In 2013, the standard IYCF interviewer-administered questionnaire was used in the Chinese National Nutrition and Health Survey (CNNHS) to assess the status of infant and young child feeding practices in China [8]. Our study team has also used this interviewer-administered questionnaire in previous studies [12-14].

Although traditional interviewer-administered household surveys are the primary data source of coverage indicators on children in LMICs [9], they are labour-intensive, time-consuming, and costly [15]. Researchers have been exploring new ways of overcoming the survey method’s shortcomings [16,17]. Self-administered electronic questionnaires have become an important data collection tool in public health and epidemiology [18]. Compared with traditional interviewer-administered data collection methods, this method can achieve a broader population coverage, collect data quicker, and reduce the cost [18].

Self-administered electronic surveys need to be validated before being used, especially for complicated questionnaires. In 2013, we conducted a study to compare the agreement between short message service (SMS) and interviewer-administered data collection of IYCF indicators, which found poor agreement issues related to the SMS technology [12]. Moreover, few people currently use SMS to communicate [19].

With the rapid development of the Internet and new media, communication apps such as WeChat are widely used in China. Similar to Facebook, WeChat is a free social networking app that was released by Tencent in 2011. WeChat has become the most popular mobile social media platform, with 73.7% of Chinese users accessing the platform frequently [20]. It provides a variety of daily life services, including instant messaging, interest or private groups, instant information sharing, browsing content, and mobile payments [21]. In January 2021, 1.09 billion users opened the WeChat app and 330 million users made daily video calls [22]. WeChat has been used both in survey and intervention research [13,23-28]. However, there are few comparative studies on the data quality of WeChat-based self-administered data collection. This study aimed to explore the data agreement between a WeChat-based self-administered and WeChat-based interviewer-administered survey to collect IYCF information.

## METHODS

### Study design

We used a test-retest study design to compare data agreement between a WeChat-based interviewer-administered survey (reference standard) and a WeChat-based self-administered survey (novel method). The study took place in the central area of Fenxi County, Shanxi Province, China. Participants were mothers of children aged 6-23 months old. To collect data, we first sent a WeChat self-administered questionnaire on infant and young child feeding to each participating mother in the morning. Four to thirteen (mean = 6.2) hours after mothers completed the self-administered survey, we conducted an interviewer-administered survey in each mother’s home by using the same WeChat questionnaire. We compared the data agreement of each question and six key IYCF indicators. We also collected the reasons for the inconsistencies between the two methods.

### Study setting

Shanxi Province is in North China, with an area of 0.1567 million km^2^. By the end of 2019, the total population of Shanxi Province was 37 292 200, of which 40.5% was a rural population. The per capita disposable income for Shanxi residents in 2019 was 33 262 yuan (US$5149.2, at an exchange rate of 6.4596 on May 14, 2021) for urban and 12 902 yuan (US$1997.3) for rural areas [29].

Fenxi County is located in the south of Shanxi Province, with a total area of 880 km^2^ [30]. There are 8 townships and 121 villages in the county. In 2019, there were 150 522 permanent residents, of which 52.2% were a rural population. The annual per capita disposable income of Fenxi County was 29 024 yuan (US$4493.2) for urban areas and 4962 yuan (US$768.2) for rural areas [31]. There were 926 live births in Fenxi County in 2020 according to the Fenxi County Maternal and Child Health and Family Planning Service Center annual report (unpublished data).

### Participants

Mothers of children aged 6-23 months old in the central area of Fenxi County were invited to participate in this study. We excluded mothers if they: 1) did not register their mobile phone number; 2) did not live in the central area of Fenxi County at the time of the survey; 3) were not at home for a long time; 4) refused to participate in the survey.

### Sample size calculation

The sample size calculation for this study was based on the Cohen's kappa test that was used for the method agreement analysis [32]. Based on our previous text messaging survey [12], we estimated kappa to be 0.45, with a 0.15 confidence interval. With a power of 80% and a 5% significance level, we determined that a sample size of 269 was needed for this study. To compensate for loss to follow-up, we planned to enrol all the eligible mothers of children aged 6-23 months old living in the central area of Fenxi County.

### WeChat self-administered questionnaire development

Our WeChat self-administered questionnaire was produced and distributed using the online survey tool ‘Sojump’ (http://www.sojump.com), which is the largest free professional online survey platform in China. We set up our questionnaire on the Sojump platform, and then got a link to the electronic questionnaire from the platform. We sent the questionnaire link to participants through WeChat. Participating mothers could click the link and answer the questions. When mothers submitted the questionnaires, we could see and download the questionnaire data on the Sojump platform.

The questionnaire was designed based on the adapted WHO Maternal, Newborn and Child Health household survey (MNCHHHS) (unpublished, 2009) and “Indicators for assessing infant and young child feeding practices” (WHO&UNICEF) [11] to collect data on infant and young child feeding knowledge and practices, which used for several times in our previous studies [12-14]. There were 43 questions in the questionnaire (see the **Online Supplementary Document**), including 7 questions on basic information, 36 questions on breastfeeding and complementary feeding knowledge and practices, and complementary feeding information sources.

### Pilot testing

The WeChat self-administered questionnaire was pre-tested in Fenxi County in March 2021. Six caregivers, including five mothers and one father, were invited to fill in the WeChat self-administered questionnaire. After they completed it, we interviewed each of them by telephone and collected their feedback and suggestions on our questionnaire. We asked them whether they understood each question and encouraged them to give suggestions on how to make the questions easier to understand.

For the interviewer-administered survey, we used the same online questionnaire. Our research team sent the questionnaires to the interviewers via WeChat. The interviewers then invited mothers to complete the informed consent form and questioned the mothers following the instructions on the WeChat questionnaire by using their own smartphones.

### Training of Interviewers

Six staff from the Fenxi County Maternal and Child Health and Family Planning Service Centre were recruited as interviewers to collect data from participants. The inclusion criteria were as follows: 1) female; 2) undertook work on maternal and child health; and 3) had experience in fieldwork. We provided them with one day of training before the survey. The training course included communication skills, questionnaire explanation, role-play, and collecting reasons for inconsistencies. Members of the study team (AL and ZJ) who had experience in program management were the survey supervisors.

### Recruitment and data collection

We carried out the surveys from March 29 to April 30, 2021. Before recruitment, we asked the Fenxi County Maternal and Child Health and Family Planning Service Centre to provide a list of names of all children aged 6-23 months who lived in the central area of Fenxi County. The name list included children's names, gender, birth date, home address, parents' names and mobile phone numbers. There was a total of 434 children on the list.

### Self-administered survey

Based on the name list, the interviewers first made an appointment with mothers to be surveyed the next day by phone, and then added the mothers as WeChat friends.

On the morning of the survey day, the interviewers first sent a message to mothers to introduce our study and told them how to fill out the questionnaire through WeChat. After that, the interviewers sent the WeChat self-administered questionnaire with informed consent to each mother. Once a mother submitted the questionnaire, the research team could see the data immediately on the Sojump platform. For the mothers who did not complete the questionnaire before 10 am, the interviewers reminded them once.

### Interviewer-administered survey

Four to thirteen (mean = 6.2) hours after mothers completed the WeChat self-administered questionnaires, the interviewers went to each mother’s home and conducted the interviewer-administered survey to collect the same information through the same WeChat questionnaire. Once an interviewer submitted the interviewer-administered questionnaire, a supervisor (ZJ) immediately downloaded the data of the two survey methods for the same mother from the “Sojump” platform and manually compared the agreement for each question. The supervisor (ZJ) informed the interviewer about all inconsistent questions and their corresponding answers via WeChat messages. The interviewer then asked the mothers why they provided different answers in the two surveys and wrote down all the reasons the mothers gave in an interview’s daily record form.

### Main outcomes

The main outcome of the study was data agreement, which was defined as the agreement between the WeChat self-administered questionnaire and the interview-administered questionnaire, including all questions and six key IYCF indicators [11] (**Box 1**).

Box 1 **WHO key IYCF indicators/**

1. Minimum dietary diversity: the proportion of children aged 6-23 months who receive foods from four or more food groups was estimated. The seven food groups used for calculation of this indicator were: 1) grains, root and tubers; 2) legumes and nuts; 3) dairy products (milk, yogurt, cheese); 4) meat (meat, fish, poultry and liver/organ meat); 5) eggs; 6) vitamin-A rich fruits and green vegetables; 7) other fruits and vegetables.

2. Minimum meal frequency: the proportion of breastfed and non-breastfed children aged 6-23 months who received solid, semi-solid, or soft foods (also including milk for non-breastfed children) the minimum number of times or more.

3. Minimum acceptable diet: the proportion of children aged 6-23 months who reached a minimum dietary diversity and minimum meal frequency.

4. Consumption of iron-rich or iron-fortified foods: the proportion of children aged 6-23 months who received iron-rich food or iron fortified food that was specially designed for infants and young children, or that was fortified in the home.

5. Continued breastfeeding at 1 year: the proportion of children 12-15 months of age who were fed breast milk.

6. Continued breastfeeding at 2 years: the proportion of children 20-23 months of age who were fed breast milk.

The secondary outcome was the reasons for the inconsistencies between the survey methods for each question. All the reasons for inconsistencies collected after the interviewer-administered surveys were entered in Excel by the two supervisors (AL and ZJ). There were 523 reasons which were divided into three categories and ten sub-categories. First, there were errors caused by self-administered methods: mothers not understanding the questions; mothers not answering carefully; and mothers’ operating errors. Second, there were errors caused by interviewer-administered method: interviewers not explaining the questions clearly; interviewers’ operating errors; mothers giving wrong answers due to nervousness when facing the interviewers; and mothers not answering carefully. Third, there were errors caused by reasons unrelated to both methods: mothers forgetting what the child ate; mothers changing their mind; and mothers having difficulty understanding questions in both surveys. We counted the frequency and calculated the percentages.

In addition, we also compared the differences between the two methods for time consumption and monetary costs. For the WeChat self-administered survey, the time consumption referred to the time that mothers spend filling in the questionnaires. For the interviewer-administered survey, the time consumption included the time of travel for the interviewers and filling in the questionnaires. The Sojump platform provided the duration of data recording for both methods. We asked the interviewers to record the duration for each visit.

The costs for both survey methods included gifts for the participants, accident insurance, labour costs, and travel costs for the interviewers. We sent each participant a complementary food book as a gift, which was 24 yuan (US$3.7). We allocated 12 yuan (US$1.7) as gifts’ cost for both survey methods. The accident insurance for each interviewer was 20 yuan (US$3.1). For the labour costs, we gave interviewers 7 yuan (US$1.1) for each WeChat self-administered questionnaire, as they had to send the WeChat self-administered questionnaires to mothers and remind them to complete them, and 28 yuan (US$4.3) for each interviewer-administered questionnaire. For the travel costs of the interviewer-administered survey, we asked the interviewers to record the transportation fee of each trip.

### Statistical analysis

Questionnaire data uploaded to the Sojump platform were automatically converted into a Microsoft Excel sheet. After the data cleaning, we converted the Excel sheet into a database file (dbf) for the final analysis.

We used SPSS version 26 (IBM SPSS Statistics, IBM Corporation, Somers, NY, USA) for the statistical analysis. The median (interquartile range) was used to describe in continuous variables. Percentages are used to present categorical variables.

We assessed data agreement by using Cohen's kappa score (K) values (simple k for categorical variable) and intraclass correlation coefficient (ICC, for continuous variables) for both methods. Kappa and ICC values have the following meaning: <0.0 = poor; 0.00-0.20 = slight; 0.21-0.40 = fair; 0.41-0.60 = moderate; 0.61-0.80 = substantial; and 0.81-1.00 = almost perfect [32]. The percentage of “agreement” was defined as the number of mothers who gave the same answers for each question divided by the total number of participants.

We used the McNemar’s test for categorical variables and the Wilcoxon Test for continuous variables to detect differences between survey methods in each question, as well as the IYCF indicators. *P* – values less than 0.05 were considered as statistically significant.

### Ethical considerations

The study was approved by the Ethical Committee of the Capital Institute of Pediatrics in Beijing. All interviewees read the Information Sheet and provided both electronic and written informed consent. There was an electronic informed consent in each WeChat self-administered questionnaire, and participating mothers read the informed consent and clicked “Agree to participate” before they answered the questions. In the interviewer-administered survey, the interviewers showed and explained the paper informed consent to mothers and obtained oral and written informed consent.

## RESULTS

**Figure 1:**
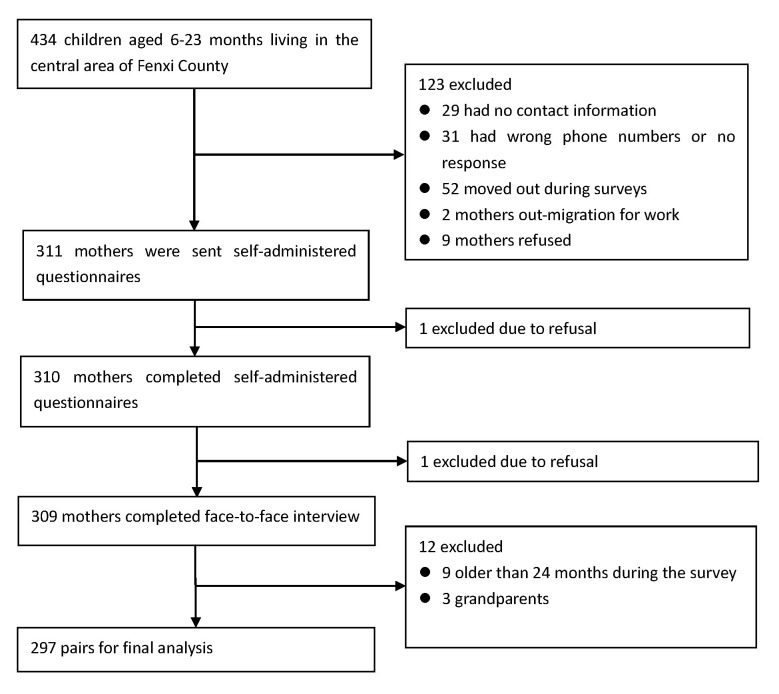
Flow of study participants.

There were 434 children aged 6-23 months living in the central area of Fenxi County in February 2021. We had to exclude 123 children because we could not contact their mothers, they had moved out, or their mothers were unavailable. A total of 309 mothers completed both the self-administered and interviewer-administered questionnaires. Nine mothers whose children were older than 24 months during the survey and three grandparent respondents were excluded, leaving 297 mothers for the final analysis. The flow of the study participants is displayed in **Figure 1**.

**Table 1** lists demographic characteristics of mothers and their children. Girls accounted for 53.9% (160/297) and two-thirds of children were aged 12-23 months. The median age of mothers was 31, and they were generally well educated, with 70% (208/297) of them attending senior high school or above and only 1% (3/297) attending primary school or below.

**Table 1:** Characteristics of children and their mothers (n = 297)

Characteristics of participants	No. of participants	Percentage (%)
**Children:**		
Gender:		
Boy	137	46.1
Girl	160	53.9
Age in months		
6-11	99	33.3
12–23	198	66.7
**Mothers:**		
Median age in years (Q1–Q3)	31	29, 33
Education:		
Primary school or below	3	1.0
Junior high school	86	29.0
Senior high school	100	33.7
College	105	35.3
Graduate or above	3	1.0

**Table 2** shows data agreement of the answers to the survey questions between the two methods.

**Table 2:** Data agreement of questions between the two methods (n = 297)

Questions	Self-administered (No., %)/ (Median, IQR)	Interviewer-administered (No., %)/ (Median, IQR)	Agreement (No., %)	κ/ICC (95% CI)	κ/ICC values	*P-*value for κ/ICC	*P-v*alue for McNemar/ Wilcoxon Test
**Feeding knowledge**							
Q1: Can you tell me until what age a baby should only receive breastmilk? (months)	6 (4, 6)	6 (5, 6)	260 (87.5)	0.71 (0.65, 0.76)*	Substantial	<0.001	0.15†
Q2: Can you tell me until what age a baby should start receiving foods such as mashed or solid foods? (months)	6 (6, 7)	6 (6, 7)	256 (86.2)	0.003 (-0.11,0.12)*	slight	0.48	0.28†
Q3: Can you tell me until what age a baby should be breastfed? (months)	12 (12, 20)	12 (12, 20)	270 (90.9)	0.86 (0.82, 0.88)*	almost perfect	<0.001	0.48†
**Feeding practices**							
Q4: Has your child ever been breastfed?	283 (95.3)	285 (96.0)	291 (98.0)	0.76 (0.71, 0.80)	Substantial	<0.001	0.69
Q5: Has your child ever been fed solid or semi-solid food?	285 (96.0)	281 (94.6)	289 (97.3)	0.70 (0.64, 0.76)	Substantial	<0.001	0.29
Q6: At what age did you first feed your child her/his first solid and semi-solid food?	6 (6, 7)	6 (6, 7)	251 (84.5)	0.23 (0.22, 0.42)*	Fair	<0.001	0.64†
Q7: Did you breastfed your child yesterday during the day or at night?	163 (54.9)	162 (54.5)	289 (97.3)	0.95 (0.94, 0.96)	almost perfect	<0.001	1.00
Q8: Did your child drink plain water / mineral water / sugar water / tea yesterday during the day or at night?	271 (91.2)	270 (90.9)	286 (96.3)	0.77 (0.72, 0.81)	substantial	<0.001	1.00
**Yesterday during the day or night, did your child drink/eat any the following food group items?**							
Q9: Grains (Porridge, bread, rice, noodles, or other foods made from grains)	258 (86.9)	256 (86.2)	279 (93.9)	0.74 (0.68, 0.79)	substantial	<0.001	0.82
Q10: Roots (White potatoes, white yams, cassava)	51 (17.2)	46 (15.5)	288 (97.0)	0.89 (0.86, 0.91)	almost perfect	<0.001	0.18
Q11: Sweet potatoes that are yellow or orange inside	46 (15.5)	46 (15.5)	291 (98.0)	0.92 (0.90, 0.94)	almost perfect	<0.001	1.00
Q12: Any dark green leafy vegetables	161 (54.2)	165 (55.6)	281 (94.6)	0.89 (0.87, 0.91)	almost perfect	<0.001	0.45
Q13: Pumpkin, carrots, tomato, mango that are yellow or orange inside	130 (43.8)	118 (39.7)	265 (89.2)	0.78 (0.73, 0.82)	Substantial	<0.001	0.05
Q14: Any other vegetables and fruits	225 (75.8)	228 (76.8)	276 (92.9)	0.81 (0.76, 0.84)	almost perfect	<0.001	0.66
Q15: Any meat, such as beef, pork, lamb, goat, chicken, or duck	119 (40.1)	127 (42.8)	287 (96.6)	0.93 (0.92, 0.95)	almost perfect	<0.001	0.02
Q16: Fish, shrimp or other seafood	22 (7.4)	21 (7.1)	294 (99.0)	0.93 (0.91, 0.94)	almost perfect	<0.001	1.00
Q17: Liver, kidney, heart, or other organ meats	6 (2.0)	7 (2.4)	294 (99.0)	0.76 (0.71, 0.81)	substantial	<0.001	1.00
Q18: Eggs	257 (86.5)	259 (87.2)	287 (96.6)	0.85 (0.82, 0.88)	almost perfect	<0.001	0.75
Q19: Any foods made from beans, peas, lentils	105 (35.4)	180 (60.6)	273 (91.9)	0.83 (0.79, 0.86)	almost perfect	<0.001	0.02
Q20: Any nuts or seeds	42 (14.1)	46 (15.5)	283 (95.3)	0.81 (0.77, 0.85)	almost perfect	<0.001	0.42
Q21: Infant formula	185 (62.3)	180 (60.6)	284 (95.6)	0.91 (0.89, 0.93)	almost perfect	<0.001	0.27
Q22: Milk	16 (5.4)	19 (6.4)	292 (98.3)	0.85 (0.81, 0.88)	almost perfect	<0.001	0.38
Q23: Cheese, yogurt, or other milk products	27 (9.1)	21 (7.1)	285 (96.0)	0.73 (0.67, 0.78)	substantial	<0.001	0.15
Q24: How many times did your child drink infant formula yesterday during the day or at night?	1 (0, 3)	1 (0, 3)	267 (89.9)	0.77 (0.72, 0.82)*	substantial	<0.001	0.17†
Q25: How many times did your child drink milk yesterday during the day or at night?	0 (0, 0)	0 (0, 0)	289 (97.3)	0.30 (0.19, 0.40)*	fair	<0.001	0.89†
Q26: How many times did your child eat solid, semi-solid, or soft foods other than liquids yesterday during the day or at night?	2 (1, 3)	2 (1, 3)	251 (84.5)	0.63 (0.56, 0.70)*	substantial	<0.001	0.02†
Q27: Yesterday, during the day or night, did your child consume any food to which you added a powder or sprinkles contained iron, or any lipid that contains iron?	143 (48.1)	150 (50.5)	272 (91.6)	0.83 (0.79, 0.86)	almost perfect	<0.001	0.23
**Information sources:**							
Q28: Have you received any complementary feeding information before and after delivery	194 (65.3)	191 (64.3)	276 (92.9)	0.90 (0.87, 0.92)	almost perfect	<0.001	0.62
Q29: From family members, relatives, friends or neighbors	97 (32.7)	102 (34.3)	290 (97.6)	0.95 (0.94, 0.96)	almost perfect	<0.001	0.13
Q30: From hospital of county or above	33 (11.1)	29 (9.8)	289 (97.3)	0.86 (0.82, 0.88)	almost perfect	<0.001	0.29
Q31: From township hospital or community health center	111 (37.4)	108 (36.4)	282 (95.0)	0.89 (0.87, 0.91)	almost perfect	<0.001	0.61
Q32: From village clinics or community health stations	43 (14.5)	46 (15.5)	288 (97.0)	0.88 (0.85, 0.90)	almost perfect	<0.001	0.51
Q33: From private hospitals or clinics	6 (2.0)	7 (2.4)	296 (99.7)	0.92 (0.90, 0.94)	almost perfect	<0.001	1.00
Q34: Internet (Baidu, Sogou, Wechat account, microblog)	92 (31.0)	93 (31.3)	286 (96.3)	0.91 (0.89, 0.93)	almost perfect	<0.001	1.00
Q35: From traditional mass media (newspaper, magazine, TV, books)	30 (10.1)	30 (10.1)	289 (97.3)	0.85 (0.82, 0.88)	almost perfect	<0.001	1.00
Q36: From other sources	17 (5.7)	13 (4.4)	291 (98.0)	0.79 (0.74, 0.83)	Substantial	<0.001	0.22

*ICC – intra-class correlation coefficient

†Wilcoxon test.

For three feeding knowledge questions, agreement was substantial for “Duration of exclusive breastfeeding (Q1)” (ICC = 0.71, 95% CI = 0.65-0.76), poor for “Months for introducing complimentary food (Q2)” (ICC = 0.003, 95% CI = 0.11-0.12), and almost perfect for “Duration of breastfeeding (Q3)” (ICC = 0.86, 95% CI = 0.82-0.88). Wilcoxon Test on these paired quantitative data showed no significant differences (*P* = 0.15 for Q1, *P* = 0.28 for Q2, and *P* = 0.48 for Q3, respectively).

There were 24 questions about feeding practices. For the four questions on continuous data, the agreement of two questions was substantial (ICC = 0.77 for Q24, ICC = 0.63 for Q26), and the agreement of the other two questions was fair (ICC = 0.23 for Q6 and ICC for Q25 = 0.30 for Q25). Wilcoxon Test showed that there was a significant difference for the one question (*P* = 0.02 for Q26) and no significant differences for the other three questions (*P* = 0.64 for Q6, *P* = 0.17 for Q24 and *P* = 0.89 for Q25, respectively).

Among the 20 questions on categorical data, the agreement of 13 questions was almost perfect (κ = 0.81-0.95), and the agreement of the other 7 questions was substantial (κ = 0.63-0.78). The McNemar Test showed that there were significant differences for Q15 (*P* = 0.21) and Q19 (*P* = 0.23), and no significant differences for the other 18 questions.

All nine questions on mothers' complementary feeding information received and sources were categorical data, and the agreement was almost perfect (K ≥ 0.80).

**Table 3** demonstrates data agreement of key IYCF indicators between the two methods. The agreement for “Minimum dietary diversity”, “Consumption of iron–rich or iron fortified foods”, “Continued breastfeeding at 1 year” and “Continued breastfeeding at 2 years”, was almost perfect (κ = 0.84-0.94), while the agreement for “Minimum meal frequency” and “Minimum accepted diet” was substantial (κ = 0.78 and κ = 0.80, respectively). Moreover, the proportion of “Minimum meal frequency” and “Minimum accepted diet” showed statistical differences between the two survey methods (*P* = 0.03 and *P* = 0.001, respectively).

**Table 3:** Data agreement of key IYCF indicators between the two methods

Key IYCF indicators	No. of pairs	Self-administered (No., %)	Interviewer-administered (No., %)	Agreement (No., %)	κ (95% CI)	Κ values	*P-*value for kappa	*P-*value for McNemar Test
Minimum dietary diversity	297	230 (77.4)	232 (78.1)	285 (96.0)	0.88 (0.86, 0.91)	almost perfect	<0.001	0.77
Minimum meal frequency	297	173 (58.2)	186 (62.6)	266 (89.6)	0.78 (0.73, 0.82)	substantial	<0.001	0.03
Minimum accepted diet	297	138 (46.5)	156 (52.5)	267 (89.9)	0.80 (0.75, 0.84)	substantial	<0.001	0.001
Consumption of iron–rich or iron fortified foods	297	200 (67.3)	209 (70.4)	276 (92.9)	0.84 (0.80, 0.87)	almost perfect	<0.001	0.08
Continued breastfeeding at 1 y	55 *	38 (69.1)	38 (69.1)	53 (96.4)	0.92 (0.86, 0.95)	almost perfect	<0.001	1.00
Continued breastfeeding at 2 y	59†	10 (16.9)	11 (18.6)	59 (100)	0.94 (0.91, 0.97)	almost perfect	<0.001	1.00

CI – confidence interval, IYCF – infant and young child feeding, κ-kappa, y – year

*The age range of this indicator (continued breastfeeding at 1 y) is relatively narrow, and only infants aged 12-15 months can be included in the calculation. In this study, 55 out of 297 people met the criteria.

†The age range of this indicator (continued breastfeeding at 2 y) is relatively narrow, and only infants aged 20-23 months can be included in the calculation. In this study, 59 out of 297 people met the criteria.

**Table 4** illustrates different reasons for inconsistencies between the two methods. There were 523 inconsistent results, and their reasons were divided into three categories: caused by self-administered method (56.4% (295/523)), caused by interviewer-administered method (10.0% (52/523)) and unrelated to both methods (33.6% (176/523)). More than half of the inconsistencies were caused by the self-administered method, which consisted of mothers' wrong operation (12.2% (64/297)), mothers' misunderstanding of the question (18.8% (98/523)) and mothers' errors (25.4% (133/523)). Inconsistencies caused by the interviewer-administered method accounted for only 10% (52/523), and most of them were due to interviewers’ wrong operation (7.3% (38/523)). About one-third of the inconsistencies were not related to the survey methods, which included mothers’ memory, changing their minds and difficulty with understanding the questions.

**Table 4:** Reasons for inconsistencies between the two methods

Classification of inconsistencies	No. of inconsistent questions	Percentage (%)
**Caused by self-administered method**	**295**	**56.4**
Mothers’ operation errors	64	12.2
Mothers did not understand the question	98	18.8
Mothers did not answer carefully	133	25.4
**Caused by interviewer-administered method**	**52**	**10.0**
Interviewers’ operation errors	38	7.3
Interviewers did not explain the questions clearly	7	1.3
Mothers did not answer carefully	5	1.0
Mothers gave wrong answers due to nervousness when facing the interviewers	2	0.4
**Unrelated to both methods**	**176**	**33.6**
Mothers forgot what the child ate and gave different answers in the two surveys	114	21.8
Mothers changed their mind	43	8.2
Mothers had difficulty in understanding questions in both surveys	19	3.6
**Total**	**523**	**100.0**

**Table 5** explains the reasons for questions and indicators with poor agreement in **Table 2** and **Table 3**. There were 41 mothers who gave reasons for inconsistent data for Q2, 46 for Q6, 10 for Q15 and 24 data for Q19; 45%-80% of them were caused by self-administered method.

**Table 5:** Causes of questions and indicators with poor agreement

Questions and indicators with poor agreement	Caused by self-administered method (No., %)	Caused by interviewer-administered method (No., %)	Unrelated to either method (No., %)	Total (No., %)
Q2: Can you tell me until what age a baby should start receiving foods such as mashed or solid foods? (months)	21 (51.2)	4 (9.8)	16 (39.0)	41 (100.0)
Q6: At what age did you first feed your child her/his first solid and semi-solid food?	21 (45.7)	5 (10.9)	20 (43.5)	46 (100.0)
Q15: Any meat, such as beef, pork, lamb, goat, chicken, or duck eaten yesterday	8 (80.0)	0 (0.0)	2 (20.0)	10 (100.0)
Q19: Any foods made from beans, peas, lentils	14 (58.3)	0 (0.0)	10 (41.7)	24 (100.0)
Minimum meal frequency *	**55 (59.8)**	**6 (6.5)**	**31 (33.7)**	**92 (100.0)**
Q7: Did you breastfed your child yesterday during the day or at night?	4 (50.0)	2 (25.0)	2 (25.0)	8 (100.0)
Q24: How many times did your child drink infant formula yesterday during the day or at night?	17 (56.7)	1 (3.3)	12 (40.0)	30 (100.0)
Q25: How many times did your child drink milk yesterday during the day or at night?	4 (50.0)	1 (12.5)	3 (37.5)	8 (100.0)
Q26: How many times did your child eat solid, semi-solid, or soft foods other than liquids yesterday during the day or at night?	30 (65.2)	2 (4.3)	14 (30.05)	46 (100.0)
**Minimum dietary diversity†**	**116 (59.2)**	**24 (12.2)**	**56 (28.6)**	**196 (100.0)**
Q9: Grains (Porridge, bread, rice, noodles, or other foods made from grains)	10 (55.5)	5 (27.8)	3 (16.7)	18 (100.0)
Q10: Roots (White potatoes, white yams, cassava)	5 (55.6)	0 (0)	4 (44.4)	9 (100.0)
Q11: Sweet potatoes that are yellow or orange inside	3 (50.0)	1 (16.7)	2 (33.3)	6 (100.0)
Q12: Any dark green leafy vegetables	10 (62.5)	1 (6.3)	5 (31.2)	16 (100.0)
Q13: Pumpkin, carrots, tomato, mango that are yellow or orange inside	16 (50.0)	6 (18.7)	10 (31.3)	32 (100.0)
Q14: Any other vegetables and fruits	15 (71.4)	2 (9.5)	4 (19.1)	21 (100.0)
Q15: Any meat, such as beef, pork, lamb, goat, chicken, or duck	8 (80.0)	0 (0)	2 (20.0)	10 (100.0)
Q16: Fish, shrimp or other seafood	1 (33.3)	1 (33.3)	1 (33.4)	3 (100.0)
Q17: Liver, kidney, heart, or other organ meats	0 (0)	2 (66.7)	1 (33.3)	3 (100.0)
Q18: Eggs	7 (70.0)	0 (0)	3 (30.0)	10 (100.0)
Q19: Any foods made from beans, peas, lentils	14 (58.3)	0 (0)	10 (41.7)	24 (100.0)
Q20: Any nuts or seeds	6 (42.9)	1 (7.1)	7 (50.0)	14 (100.0)
Q21: Infant formula	11 (84.6)	1 (7.7)	1 (7.7)	13 (100.0)
Q22: Milk	2 (40.0)	1 (20.0)	2 (40.0)	5 (100.0)
Q23: Cheese, yogurt, or other milk products	8 (66.7)	3 (25.0)	1 (8.3)	12 (100.0)
**Minimum accepted diet‡**	**171 (59.4)**	**30 (10.4)**	**87 (30.2)**	**288 (100.0)**

*The indicator (Minimum meal frequency) was calculated from the following four questions: Q7, Q24, Q25 and Q26.

†The indicator (Minimum dietary diversity) was calculated from the following fifteen questions: Q9 ~ Q23.

‡The indicator (Minimum accepted diet) was calculated from the following two indicators: Minimum meal frequency and Minimum dietary diversity.

For all 3 indicators, just under 60% of the reasons for inconsistencies were caused by self-administered method.

**Table 6** shows costs and time for the two methods. The cost of interviewer-administered survey was much higher than that of self-administered survey: ¥13 626.7 (US$2016.7) vs ¥5843 (US$873.6) for total cost, and ¥45.9 (US$6.8) vs ¥19.7 (US$2.9) for per questionnaire cost. It took the interviewers in interviewer-administered survey 19.6 minutes per questionnaire, which mainly included travel and interview time. The interviewer time for the self-administered survey was very small and could be ignored. Participants took on average 10.1 minutes to complete the self-administered and 4.5 minutes to complete the interviewer-administered survey.

**Table 6:** Costs of the two methods (n = 297)

Cost items	Self-administered		Interviewer-administered	
**Monetary cost (US$)**	**Total cost**	**Per person**	**Total cost**	**Per person**
Gift to mothers	551.7	1.9	551.7	1.9
Insurance for interviewers	0	0	18.6	0.1
Payment to interviewers	321.9	1.1	1287.4	4.3
Travel	0	0	158.9	0.5
**Total**	873.6	2.9	2016.7	6.8
**Time cost (minutes)**				
For interviewers				
Filling in questionnaires	0	0	1323	4.5
Travel	0	0	4484	15.1
**Total**	0	0	5807	19.6
**For participants**	3003	10.1	1323	4.5

## DISCUSSION

### Principal results

We compared data agreement and costs of the self-administered and interviewer-administered data collection methods for a survey on infant and young child feeding. Data agreement, which shows the quality of measurement, is crucial for a new data collection method to be accepted and can be measured comparing the differences between data collection methods [33,34]. Most of the questions in our survey showed very good agreement, of 36 questions, only 3 questions had k less than 0.6, but these questions could still be used for calculating indicators at a population level. All the six key IYCF indicators had substantial or almost perfect agreement. The cost of the interviewer-administered survey was much higher than that of the self-administered survey.

### Comparison with prior work

The agreement results of our study are in line with the previous studies [18,35-38], which we searched and identified from PubMed by using the keywords “self-administered questionnaire”, “agreement” and “Interview-Administered”. Over the past years, many studies also found a high agreement between the self-administered and interviewer administered methods [35-37]. In 2015, a systematic review showed that self-administered surveys had excellent agreement in data collection compared with interviewer-administered surveys [18]. In 2018, a study compared dietary supplement use reported on self-administered vs interviewer-administered 24-hour recalls, which proved that there were few differences in reported supplement use by mode of administration [38]. The proportion of supplement use reported by self-administered vs interviewer-administered was 46% and 43%, respectively [38].

In 2013, we conducted a study to compare the agreement of IYCF data collected by SMS and pen-and-paper to explore the feasibility of using SMS to collect information on IYCF practices in Zhao County, Hebei Province, China [12]. The data agreement for 13 questions was generally not satisfactory: almost perfect (κ = 0.81) for only 1 question, fair for 3 questions (κ was between 0.41 and 0.60), and slight for 9 questions (κ = <0.4). Three out of the six key IYCF indicators had significant differences between the methods (“Minimum dietary diversity”, “Minimum accepted diet”, “Consumption of iron–rich or iron fortified foods”). The results of the current study showed a great improvement in data agreement, and the overall agreement of WeChat self-administered survey on six key IYCF indicators (κ/ICC> = 0.80) was much higher than that of SMS survey (κ/ICC = 0.01-0.40). Two reasons may explain the better agreement that we found in the current study: 1) a WeChat questionnaire has no word limit and can provide a detailed explanation for each question, while the limited number of words resulted in some of questions being misunderstood by participants in the SMS survey; 2) participants in the previous study came from rural areas and had lower education than participants in the current study [12].

Our study also found that agreement of categorical variables was generally very good, while the agreement of continuous variables was varied. The Cohen’s kappa value of all 29 categorical variables exceeded 0.70, but three of the seven continuous variables were below 0.60. Our results are similar to Sahoo's study: among 35 categorical variables, agreement was perfect, almost perfect and substantial for 74% (n = 26), moderate, fair and slight for 26% (n = 9) of variables. However, among the five continuous variables, agreement was almost perfect for only 20% (n = 1), and poor for 80% (n = 4) [39]. The difference between the two types of variables may be due to those categorical variables having clear answers (yes or no), but continuous variables requiring numerical answers (number of months or times).

The interviewer-administered survey was used as the reference method in our study, therefore, any data inconsistencies were attributed to the self-administered method. However, our analysis of reasons found that 43.6% (228/523) of all the inconsistencies were not caused by self-administered survey. Some inconsistencies were caused by interviewers, such as interviewers did not explain the questions clearly or interviewers’ operating errors. Previous studies have indicated that the presence of an interviewer can be distracting to respondents, while a self-administered survey avoids this source of bias [40]. Moreover, some reasons were unrelated to either method, such as mothers forgetting what the child ate and giving different answers in the two survey methods, or changing their mind.

The interviewer-administered data collection is a classic and commonly used method, but it is often expensive because it usually includes various costs: labour (salary expenses of investigators during the research period), logistics (printing and transportation of paper questionnaire), travel and accommodation expenses of researchers [15,41,42]. Our study showed that the self-administered method can decrease the survey cost because it does not involve travel and accommodation costs. The cost of our self-administered survey was ¥19.7 (US$2.9) per questionnaire vs ¥45.9 (US$6.8) for the interviewer administered survey.

### Strengths and limitations

This study compared the data agreement of the two survey methods, and analysed reasons of inconsistencies, which enabled us to better evaluate the two methods. However, our study also has some limitations. First, this evaluation study took place only in one county in China, and participants were restricted to mothers who were living in the central area of the county and might have higher education than those living in rural areas and other main caregivers, and could better understand the survey questions and were more skilful at using WeChat. Therefore, caution is needed when generalizing the findings from this study to other settings. Second, the time interval between two methods was relatively short, and some participants might have still remembered the answers they gave in the first survey.

## CONCLUSIONS

This study demonstrates that most questions and IYCF indicators had very good agreement and had no statistical differences when comparing WeChat self-administered survey and WeChat interviewer-administered survey. Four key IYCF indicators, “Minimum dietary diversity”, “Consumption of iron–rich or iron fortified foods”, “Continued breastfeeding at 1 year” and “Continued breastfeeding at 2 years”, have perfect agreement. Therefore, the WeChat self-administered electronic questionnaire can be used in the future surveys when collecting data on infant and young child feeding in China.

## Additional material


Online Supplementary Document
