# Supplementary material for: Comparison of the agreement between WeChat-based self-administered and interviewer-administered data on infant and young child feeding in China: A test-retest study
Source: J Glob Health. 2022 Jun 20;12:11004. doi: 10.7189/jogh.12.11004 (PMC9210156; doi:10.7189/jogh.12.11004)
Supplement: Online Supplementary Document [file jogh-12-11004-s001.pdf]

1. ID

**ID.1**	Child name: ______________
**ID.2**	Child sex: 1.male 2.female
**ID.3**	Child birthdate（Solar calendar）:<br>__ __ __ __y__ __m__ __d
**ID.4**	Education of mother:<br>1.primary school and below<br>2.Junior high school<br>3.High school<br>4.University<br>5.graduate and above
**ID.5**	Age of monther: __ __years old
**ID.6**	Hukou of mother? 1.Urban 2.Rural
**ID.7**	Phone number: __________________

2.Breast-feeding and nutrition（BN）

**Feeding knowledge.**	
Q1:	Can you tell me until what age a baby should only receive breastmilk? (months)
	__ __months [0=less than one month, 88=Don't know]
Q2:	Can you tell me until what age a baby should start receiving foods such as mashed or solid foods? (months)
	__ __months [0=less than one month, 88=Don't know]
Q3:	Can you tell me until what age a baby should be breastfed? (months) __ __m
	__ __months [0=less than one month, 88=Don't know]
**Feeding practice.**	
Q4:	Has your child ever been breastfed?
	1. Yes 2. No
Q5:	Has your child ever been fed solid or semi-solid food?
	1.Yes.<br>2.No solid or semi-solid food has been fed to the child yet——> ***ship to*** **Q26**
Q6:	At what age did you first feed your child her/his first solid and semi-solid food?
	__ __ months (fill in 00 for less than 1 month)
Q7:	Did you breastfed your child yesterday during the day or at night?
	1. Yes——>How many times did you breastfeed your child during the past 24 hours? __ __ *times [88=Don't know]*<br>2. No. my child did not eat breast milk.<br>3. No. My child has been stopped breastfeeding
Q8:	Did your child drink plain water / mineral water / sugar water / tea yesterday

	during the day or at night?
	1. Yes 2. No
**Yesterday during the day or night, did your child drink/eat any the following food group items?**	
Q9: Grains (Porridge, bread, rice, noodles, or other foods made from grains)	1. Yes 2. No
Q10: Roots (White potatoes, white yams, cassava)	1. Yes 2. No
Q11: Sweet potatoes that are yellow or orange inside	1. Yes 2. No
Q12: Any dark green leafy vegetables	1. Yes 2. No
Q13: Pumpkin, carrots, tomato, mango that are yellow or orange inside	1. Yes 2. No
Q14: Any other vegetables and fruits	1. Yes 2. No
Q15: Any meat, such as beef, pork, lamb, goat, chicken, or duck	1. Yes 2. No
Q16: Fish, shrimp or other seafood	1. Yes 2. No
Q17: Liver, kidney, heart, or other organ meats	1. Yes 2. No
Q18: Eggs	1. Yes 2. No
Q19: Any foods made from beans, peas, lentils	1. Yes 2. No
Q20: Any nuts or seeds	1. Yes 2. No
Q21: Infant formula	1. Yes 2. No
Q22: Milk	1. Yes 2. No
Q23: Cheese, yogurt, or other milk products	1. Yes 2. No
Q24: How many times did your child drink infant formula yesterday during the day or at night?	1. Yes 2. No
Q25: How many times did your child drink milk yesterday during the day or at night?	1. Yes 2. No
Q26: How many times did your child eat solid, semi-solid, or soft foods other than liquids yesterday during the day or at night?	1. Yes 2. No
Q27: Yesterday, during the day or night, did your child consume any food to which you added a powder or sprinkles contained iron, or any lipid that contains iron?	1. Yes 2. No
**Information sources**	
Where do you get information about breastfeeding?	
Q28: Have you received any complementary feeding information before and after delivery	1. Yes 2. No
Q29: From family members, relatives, friends or neighbors	1. Yes 2. No
Q30: From hospital of county or above	1. Yes 2. No
Q31: From township hospital or community health center	1. Yes 2. No
Q32: From village clinics or community health stations	1. Yes 2. No
Q33: From private hospitals or clinics	1. Yes 2. No
Q34: Internet (Baidu, Sogou, Wechat account, microblog)	1. Yes 2. No
Q35: From traditional mass media (newspaper, magazine, TV, books)	1. Yes 2. No
Q36: From other sources	1. Yes 2. No
